# Influence of the Unit Cell Parameters on the Thermomechanical Non-Symmetric In-Plane Shear Behavior of 2D Biaxial Braided Preform for Thermoplastic Biocomposites

**DOI:** 10.3390/polym14061117

**Published:** 2022-03-10

**Authors:** Wenqian Zhai, Damien Soulat, Xavier Legrand, Peng Wang

**Affiliations:** 1 Ensait, Gemtex, University of Lille, F-59056 Roubaix, France; wenqian.zhai@gmail.com (W.Z.); xavier.legrand@ensait.fr (X.L.); 2 Ensisa, Lpmt, University of Haute-Alsace, F-68000 Mulhouse, France; peng.wang@uha.fr; 3 University of Strasbourg, F-67081 Strasbourg, France

**Keywords:** biocomposites, thermoplastic polymer, braided preform, in-plane shear behavior, thermo-mechanics, thermoforming

## Abstract

The identification of thermomechanical in-plane shear behavior of preform is one of the most important factors to ensure the quality of the thermoplastic composites during the thermoforming process. In this present work, the non-symmetric in-plane shear behavior of flax/polypropylene 2D biaxial braided preform for thermoplastic biocomposites was characterized at elevated temperature chamber by using bias-extension test. Analytical models of a bias-extension test based on non-symmetric unit cell geometry for 2D biaxial braids were defined and applied; the thermo-condition-dependent experiments were conducted to study the temperature and displacement rate dependences. The influence of unit cell geometry parameters including braiding angle, tow waviness, and cover factor on the thermal in-plane shear behavior was deeply invested, experiments in both axial and transversal directions were performed for a complete study, and asymmetric scissor mechanisms for in-plane shear behavior were introduced and studied. Finally, a simulation of thermal impregnation distribution based on unit cell geometry was made to clarify the importance of the overall fiber volume fraction.

## 1. Introduction

Awareness of environmental and efficient issues in the last decades contributes to the important factors that encourage researchers to explore thermoplastic biocomposites [1,2,3]. In hybrid yarn-based preform, the thermoplastic resin is already hybrid with the reinforcing fiber into the continuous textile preform to form intermedia materials for a fast-manufacturing process. In comparison with thermoset preform draping, which needs a long polymerization stage in an autoclave, thermocompression of thermoplastic preform is closer to the biocomposites requirement with advantages such as: more efficient and recyclable, unlimited preform storage life, high potential in reducing cycle time, and cost consuming, and has been widely used for automotive and aeronautical industry [4,5,6]. The mechanical behavior of preforms concerns mainly biaxial tensile, bending and in-plane shear properties [6]. During the thermoforming stage, in-plane shear of the preform is the predominant deformation mode to obtain double curved shapes [7,8]. The defects such as wrinkling, buckling, porosities, misalignment, and fiber fracture, etc. [9,10,11] can be issued from specific properties of the preform (fiber strength, textile reinforcement type and geometry) or from manufacturing parameters (tool loads, blank holder, temperature, etc.) [5]. The shear angles can be large, and over a limit value which is often called the “locking angle” depending on textile reinforcement properties, the wrinkling will appear even though there is no direct relation between shear angle and wrinkling [12,13]. Once the thermoforming defects are created, they cannot be removed in the cooling stage and are consequently remained in final composites [6]. Therefore, the study on in-plane shear behavior is very important to improve the fundamental understanding of thermoforming, to provide better notice for reinforcement design and manufacturing condition decision, to avoid the defects formation.

Thermoplastic preform must be heated over the matrix melting temperature to render the textile reinforcement deformation possible. Compared to the large amount of works on the in-plane shear behavior identified in the dry-state at room temperature [14,15,16,17,18,19], the characterization at elevated temperature [5,8,20,21,22,23] is difficult but essential. Most experimental and computational work has been done on 2D-woven preform, non-crimp fabrics [24,25,26], 3D-woven interlock preform [27,28,29], or 3D-woven tufting preform [30]; while the study of the in-plane shear behavior of braided preform is limited. It is essential because with the potential to produce near-net or complex preforms at a reasonable cost and the excellent mechanical properties, such as high shear and torsional strength, increased transverse moduli, high damage resistance, and high delamination resistance, braided preform has been widely used throughout the industry [31]. Three test methods, biaxial shear test [32], uniaxial bias-extension test [33,34] and picture-frame test [35] are used to characterize the in-plane shear behavior [36]. “Bias-extension test” has several advantages: the absence of spurious tensions in the tows of the sheared zones; and the relative simplicity and its moderated size. This is very important for the test performed at elevated temperature in an oven for thermoplastic preform. “Bias-extension test” in axial or transversal directions can be applied for the study of in-plane shear of 2D biaxial braids since the braider tows are already bias interlaced and can be rotated with the existing intertow angle. Xiao et al. developed a geometric criterion and an analytical model of bias-extension test for non-orthogonally textile structure in the dry-state, experimental and analytical results presented good agreement [37]. But there is no study yet on the in-plane shear behavior of braided preform at elevated temperature chamber. 2D biaxial braided structures and sheared 2D woven structures possess similar interlacement geometry. The difference is that the intertow angles vary from 10° to 170° (braiding angles from 5° to 85°) in braiding while it remains as 90° in weaving [38,39]. The difference between 2D woven and 2D braided is compared in the previous references [40,41], once again, the influence of geometric parameters of braids [42] on in-plane shear behavior is not detailed.

Hence, the main objective of the present article is to investigate the thermomechanical in-plane shear behavior of 2D biaxial braided thermoplastic preform for biocomposites by identifying unit cell geometry parameters and studying their influences to optimize this behavior during the thermoforming stage. This experimental approach is made with bias-extension tests, which can be easily performed in a small isothermal chamber. The 2D biaxial braided thermoplastic preform is made from flax/polypropylene (PP) hybrid yarn; the general analytical model of the bias-extension test established by Xiao et al. [37] for non-orthogonally textile structure based on unit cell geometry is applied at elevated temperature for all the experiments. The experimental tests are performed at different temperatures and different displacement rates at first; this is necessary since the in-plane shear behavior is sensitive to the temperature and displacement rate due to the matrix’s thermo-dependent viscosity property [22,43,44]. Then one thermo-condition is confirmed, the thermal in-plane shear behavior is experimentally identified for different geometric parameters (braiding angle, tow waviness and cover factor) and independently in the axial (AD) and transversal (TD) directions for a complete study, thanks to the definition of the non-orthogonal and non-symmetric interlaced unit cell geometry and introduction of scissor mechanism. Influences of unit cell geometry parameters and asymmetry of AD and TD are discussed; a simulation of thermal impregnation distribution based on unit cell geometry is made. In Appendix A, the verification of the analytical model in the thermo-state is given.

## 2. Analytical Model Based on Unit Cell Geometry

### 2.1. Non-Symmetric Unit Cell Geometry of 2D Biaxial Braids

**Figure 1:**
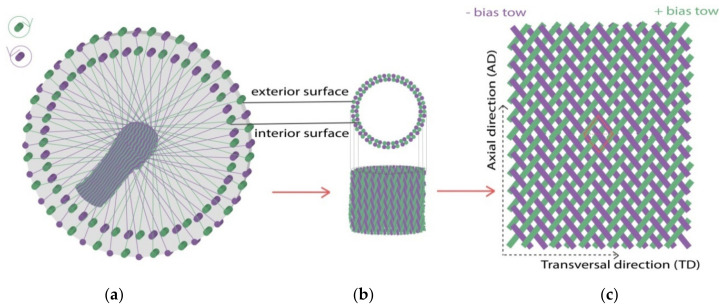
2D biaxial braids from macro-scale to meso-scale: (**a**) integral braids from loom; (**b**) circular braids part and (**c**) opened one-layer braided specimen.

2D biaxial braids, from macro-scale to meso-scale, are illustrated in Figure 1. They are manufactured on a circular braiding loom (with 96 bobbin tows carriers, for example), represented in Figure 1a. Horn gears move two sets of bobbin carriers (in green and purple colors in the illustration) in opposite directions so that the tows interlace to form biaxial braids [45,46]. Figure 1b shows the circular braids structure, which can be directly used for near-net-shape production. Half of bobbin carriers move in the outer ring of braiding loom constituting the exterior surface of braids, and the other half of the bobbin carriers move in the inner ring, constituting the interior surface of braids. Figure 1c is the specimen for the bias-extension test after opening and cutting. The Exterior surface of the tubular braids becomes the front side of the one-layer braided specimen, and the interior surface becomes the backside. The braider tows follow + β/2 and—β/2 directions and are braided in a 2 × 2 pattern in this study, that is, a + β/2 braider tow (in green color) continuously passes over two—β/2 braider tows and then under two—β/2 braider tows (in purple color) and vice versa. The rhomboid marked in red color is the unit cell.

**Figure 2:**
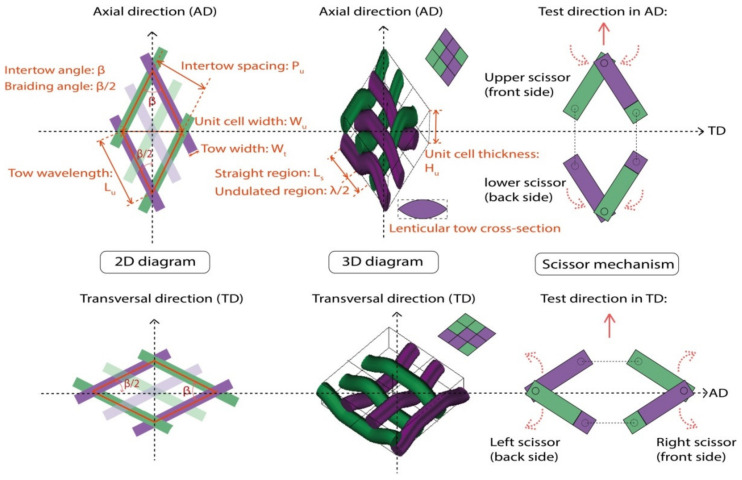
Unit cell geometry of 2D biaxial braids: test direction in AD and TD.

Unit cell geometry of a 2D biaxial braided structure consists of crimped interlacing tows in the minimum unit. Bias-extension tests should be conducted in AD and TD directions for one braid considering the non-symmetric of the braided structure. Hence, Figure 2 shows the rhomboid unit cell geometry details for the situations of test directions in AD and TD for one braid, including 2D, 3D diagrams, and scissor mechanism of in-plane shear behavior. The intertow arrangement that can be easily obtained from 2D diagram includes the intertow angle β (always in an axial direction), tow wavelength *Lu*, the intertow spacing *Pu*, and unit cell width *Wu*. From the 3D diagram with interlacement code, the undulated braider tow interlacement λ and tow cross-section (lenticular tow cross-section with a tow width *Wt*) give the unit cell a certain thickness *Hu*. The side of the rhomboid unit cell element *Lu* contains straight region *Ls* and undulated region λ/2. Three parameters are used to describe the unit cell geometry [47]: the braiding angle (BA), which is measured by ± β/2; the tow waviness ratio (WR), which in turn depends on the degree of interlacement crimp (braiding pattern and intertow spacing), is defined as Equation (1); the cover factor (*CF*), which is used to measure the braider tows deposition and defined as the percent of the mandrel surface covered by the braider tows for circular braids, is calculated by Equation (2) as the ratio of braiding tows covered area to the total rhomboid unit cell area. The overall fiber volume fraction, which depends on the tow waviness ratio and cover factor is used to specify the fiber volume fraction in the entire braids, has great effect on the thermal in-plane shear behavior.

(1) $$WR=\frac{H_{u}}{P_{u}}$$

(2) $$CF=1-\frac{\left(L_{u}-W_{t}\tan\frac{\beta }{2}-W_{t}\cot\frac{\beta }{2}\right)\left(P_{u}-2W_{t}\right)}{L_{u}P_{u}}$$

The Scissor mechanism is introduced to describe the in-plane shear behavior, which contains a scissor in the front side and an opposite scissor in the backside of the braided specimen in the unit cell. The straight region *Ls* on the *Lu* can be regarded as the scissor blade and the undulated region λ/2 can be regarded as the cross-over point. A rhomboid unit cell is composed of a pair of scissors, with the asymmetry in AD and TD. This means that an extension in AD makes upper and lower scissors inward (scissor angle decreases) and an extension in TD makes right and left scissors outward (scissor angle increases). That is why it is specified that the shear angle in the TD direction as a negative value, and it creates a negative shear moment.

### 2.2. Analytical Model of Bias-Extension Test for In-Plane Shear Behavior

Bias-extension test is performed on the rectangular textile 2D biaxial braided specimen; three assumptions are made for the identification of in-plane shear behavior:

– Tows are inextensible, or their elongation is null in the bias-extension test;
– There is no slippage between ±β/2 interlacing tows at the cross over points;
– Bending stiffness of tows is neglected.

**Figure 3:**
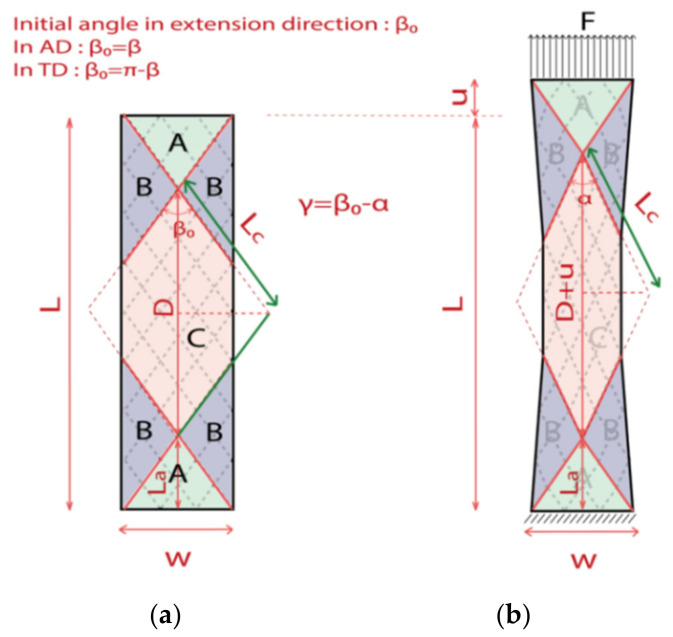
Kinematics of 2D biaxial braided specimen during the bias-extension test; (**a**) initial configuration. (**b**) during the test.

The bias-extension kinematics in initial and deformed states are shown in Figure 3. It should be noted that the 2D diagram for tests in AD and TD are both the same, for this reason, the initial angle in the extension test direction is denoted as β_0_ (10° < β_0_ < 170°). β_0_ = β for the test in AD; and β_0_ = π − β for the test in TD. Figure 3a shows the specimen dimension with length (L) along the extension direction and width (w), where the ratio r of the L and w is determined geometrically; a restrictive condition is demanded, making sure that the central zone are free at both their ends (Equation (3)). Consequently, it can be observed normally that there are 3 zones divided in the specimen along the interlacement direction in Figure 3a: undeformed zone (zone A), trellising pure shear central zone (zone C), and the zone (zone B) where the shear angle is half of that in the pure shear zone. The shear angles in each zone are constant.

(3) $$r\geq \frac{2}{\tan\left(\frac{\beta_{0}}{2}\right)}$$

where $r=\frac{2}{\tan\left(\frac{\beta_{0}}{2}\right)}$ is the case of minimum pure shear zone.

When a clamping force *F* is applied, the specimen is stretched from *L* to *L* + *u*, the rigid stiffness of the tows leads to in-plane shear deformation with no extensions of the tows. The angle β_0_ in test direction in pure shear zone decreases progressively to α, as shown in Figure 3b, generating a shear angle γ (defined as γ = β_0_-*α*). In zone B, the shear angle will be γ/2.

(4) $$\cos\left(\frac{\alpha }{2}\right)=\frac{D+u}{2L_{c}}$$

(5) $$D=L-2L_{a}=L-\frac{w}{\tan\left(\frac{\beta_{0}}{2}\right)}$$

(6) $$L_{c}=\frac{D}{2\cos\left(\frac{\beta_{0}}{2}\right)}$$

where *u* is the displacement, *D* is the diagonal of the rhombus of zone *C* in the initial condition, *L_c_* is the side of zone C, *L_a_* is the height of zone A, during the deformation, *L_c_* and *L_a_* are constant. Finally, the shear angle during the extension test can be demonstrated as Equation (7) [37].

(7) $$\gamma =\beta_{0}-2\arccos\left[\cos\left(\frac{\beta_{0}}{2}\right)+\frac{u\;sin\left(\frac{\beta_{0}}{2}\right)}{L\;tan\left(\frac{\beta_{0}}{2}\right)-w}\right]$$

**Figure 4:**
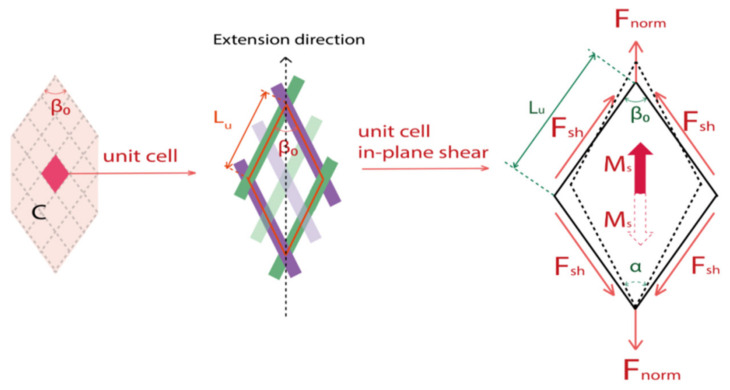
Shear load and shear moment on the unit cell.

The global load *F* measured on the tensile tester should be transformed into a quantity that considers the geometry of the specimen, denoted as normalized load *F_norm_*, which is equivalent to the value of the global load F dispersed on each unit cell. Then the shear load *F_sh_* is introduced and defined as the normalized shear tangential load per unit length along the side of the rhomboid unit cell element *L_u_* [7,48], which remains constant during in-plane shear stage as shown in Figure 4. These shear load create on the unit cell a torque *M_s_* which is the shear moment (Equation (8)).

(8) $$M_{s}\left(\gamma \right)=F_{sh}\left(\gamma \right)L_{u}cos\gamma$$

The in-plane shear virtual work, denoted *Ws*, associated to the clamping force, is dissipated in the semi-shear and pure shear zones (zone C and zone B) during the bias-extension test (Equation (9)). This gives the in-plane shear moment *M_s_* (γ) in the function of the clamping force *F* for a given shear angle [49,50] (Equations (10) and (11)).

(9) $$W_{s}=\sum_{n=1}^{ncell}\gamma M_{s}\left(\gamma \right)=F\dot{u}$$

(10) $$\sum_{n=1}^{ncell}\gamma M_{s}\left(\gamma \right)=M_{s}\left(\gamma \right)\frac{S_{C}}{S_{u}}\dot{\gamma }+M_{s}\left(\frac{\gamma }{2}\right)\frac{S_{B}}{S_{u}}\dot{\frac{\gamma }{2}}$$

(11) $$M_{s}\left(\gamma \right)=\frac{F}{\frac{S_{C}}{S_{u}}}\frac{\dot{u}}{\dot{\gamma }}-\frac{S_{B}}{2S_{C}}M_{s}\left(\frac{\gamma }{2}\right)$$

where *ncell* is the total number of unit cells, *S_B_* and *S_C_* are the initial areas of the zones B and *C*, *Su* is the surface of a unit cell, $\dot{a}$ is the rate of the quantity a. From Equation (7), the shear moment during the bias-extension test can be demonstrated as Equation (12); this equation requires an iterative procedure for its calculation as *M_s_* (γ) depends on *M_s_* (γ/2).

(12) $$M_{s}\left(\gamma \right)=\frac{F\left[L\;tan\left(\frac{\beta_{0}}{2}\right)-w\right]}{2\sin\left(\frac{\beta_{0}}{2}\right)\frac{S_{C}}{S_{u}}}\sqrt{1-{\left[cos\left(\frac{\beta_{0}}{2}\right)+\frac{u\;\sin\left(\frac{\beta_{0}}{2}\right)}{L\tan\left(\frac{\beta_{0}}{2}\right)-w}\right]}^{2}}-\frac{S_{B}}{2S_{C}}M_{s}\left(\frac{\gamma }{2}\right)$$

## 3. Materials and Experimental Set-Up

### 3.1. Materials

**Figure 5:**
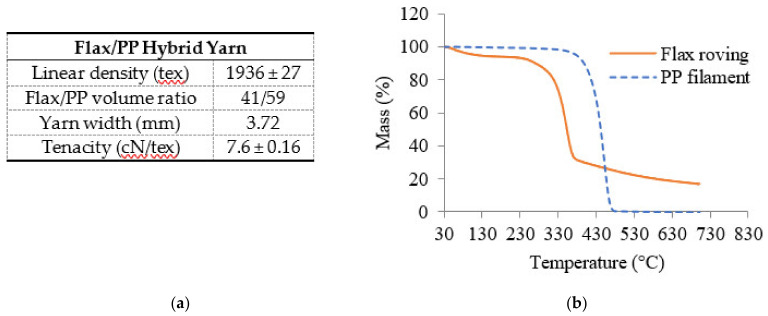
The raw material: (**a**) main properties and (**b**) TGA analysis.

The raw material for 2D biaxial braided preform was the flax/PP micro-braided hybrid yarn which was described in a previous study [51]. The main properties of hybrid yarn and thermogravimetric analysis of raw materials (flax roving and PP filament) are given in Figure 5. Regarding the flax roving, the temperatures of evaporation of absorbed moisture, the degradation of low molecular weight of hemicellulose and lignin were below 130 °C, 230–280 °C, and 325–360 °C, respectively. There was no significant mass reduction up to 430 °C for the PP filament. The melting temperature of the PP filament, 165 °C, was measured by DSC (differential scanning calorimetry).

**Figure 6:**
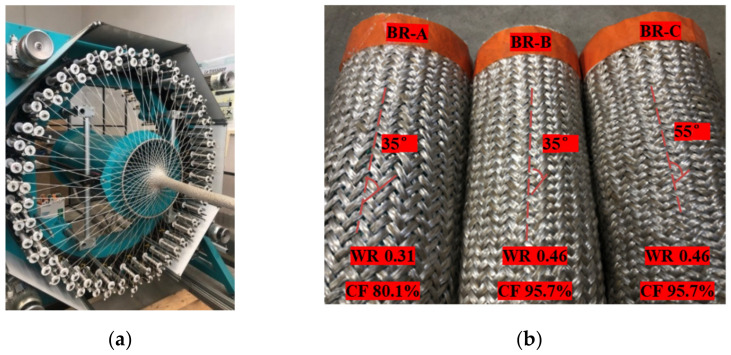
2D biaxial braided preform: (**a**) braiding loom, (**b**) obtained different 2D biaxial braids tubes.

The 2D biaxial braided prepregs were manufactured by circular braiding loom with 96 bobbin tows carriers (Herzog GLH 1/97/96-100) at Gemtex laboratory, shown in Figure 6a. Based on the unit cell geometry, braiding process parameters as production speed and carrier rotational speed were chosen to obtain three different circular braided preforms [52]: BR-A, BR-B, and BR-C, as shown as Figure 6b. BR-A and BR-B possess the same braiding angle but a different tow waviness ratio and cover factor; BR-B and BR-C possess the same tow waviness ratio and cover factor but a different braiding angle. The circular braids BR-A and BR-C are wider than BR-B to facilitate comparative study, the unit cell width of BR-A and BR-C is equal by controlling the braiding process parameters.

**Figure 7:**
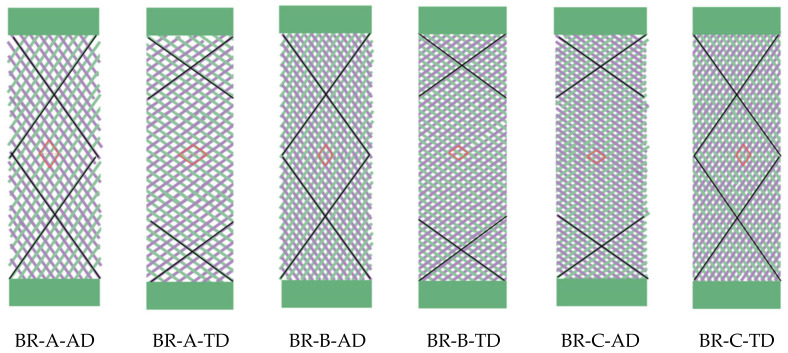
Illustrations of the six braided specimens.

After braiding, the different circular braided preform were opened and cut in AD and TD to obtain the one-layer braided specimens (with surface dimension 70 × 200 mm^2^, for which r = 2.86 respecting the restrictive condition developed as Equation (3)). The main properties of these six different braided specimens, illustrated in Figure 7, are listed in Table 1. Unit cell geometries were measured by Image J software; thickness was evaluated according to the ISO 5084 standard [53]; tow waviness ratio and cover factor were calculated by Equations (1) and (2); areal density according to the ISO 12127 standard [54]; and air permeability according to the ISO 9237 standard [55].

### 3.2. Experimental Set-Up

**Figure 8:**
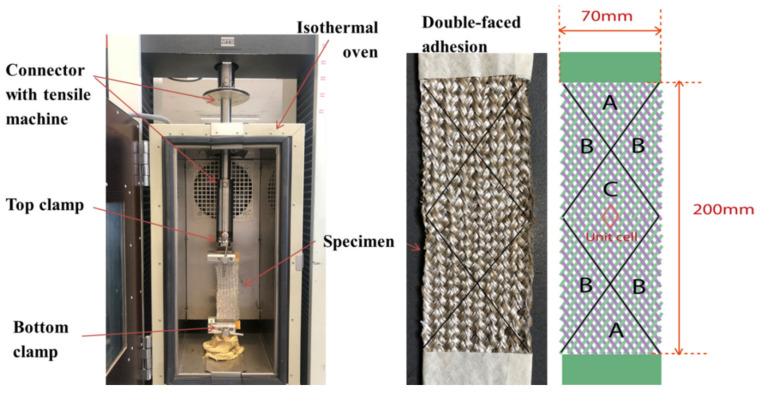
Experimental set-up for thermal bias-extension test.

All the thermal bias-extension tests were conducted using a universal tensile MTS and an isothermal oven on the specimen. Figure 8 shows the experimental setup. The specimen was inserted and fixed by two clamps. Double-faced special adhesive tape was placed at the clamped part of the specimen, avoiding the risk of slippage due to the matrix impregnation at thermo-condition. The force on the specimen was measured by a 10 kN load sensor (considering the weight of the connector). The test temperature was reached by an increasing step of 20 °C/min. Once the test temperature was reached, it needed 5 min to stabilize the specimen temperature in the oven before the bias-extension, and there were five cyclic tests.

Experiments are conducted by 2 steps. The thermo-condition dependent experiments were preliminary carried out on BR-B-AD, as a representative braided specimen. Table 2 displays the details of the tests that can be divided into two parts [56] to study the temperature (above melting temperature of PP) and displacement rate dependences. Then for the second step, a constant displacement rate of 25 mm/min and temperature at 180 °C were determined, the tests of the set of 6 specimens shown in Table 1 were performed, respectively, to study the influence of unit cell geometry on thermal in-plane shear behavior.

## 4. Results and Discussion

### 4.1. Thermo-Condition Dependent Experiments Results

**Figure 9:**
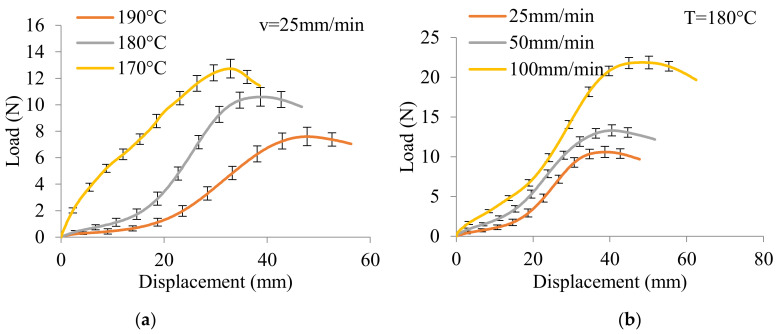
Load versus displacement curves of BR-B-AD at different thermo-conditions: (**a**) temperature-dependent experiments and (**b**) displacement rate-dependent experiments.

The load versus displacement curves of BR-B-AD (as a representative specimen) at different temperatures is presented in Figure 9a, with a constant displacement rate of 25 mm/min. The extension behavior of thermoplastic preform strongly depended on the temperature. The load was necessary to overcome the viscosity among the tows and to conduct an in-plane shear state in the beginning. Then the load increased with the displacement with weak shear stiffness until a displacement of about 20–25 mm, after which the tensile rigidity increased. The maximum load decreases and displacement increases when the temperature increases. The profile of the curve at 170 °C is different from those at 180 °C and 190 °C, because at this temperature, which is close to the melting temperature of PP, the resin started to be melted completely. The influence of displacement rate is presented in Figure 9b, with the maintained temperature at 180 °C. The curves have the same profile, showing that the increase in displacement rate, which is required relatively to the high viscosity of PP resin, led to greater load and larger displacement.

**Figure 10:**
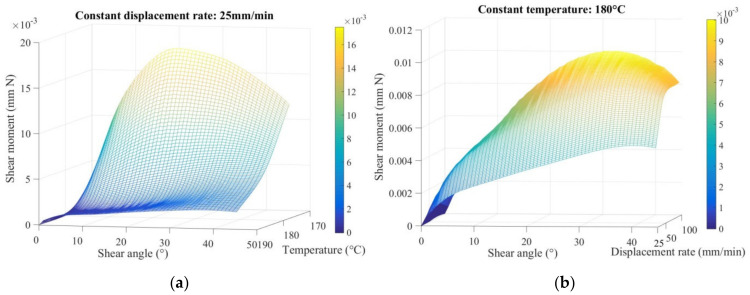
Shear moment versus shear angle meshes of BR-B-AD at different thermo-conditions: (**a**) temperature-dependent experiments and (**b**) displacement rate-dependent experiments.

The dependence of the temperature and displacement rate conditions on the in-plane shear behavior is illustrated in Figure 10 by the evolution of the shear moment as a function of the shear angle. The shear moment was calculated by Equation (12). From the mesh, there was a first stage where the shear moment increased rapidly to conduct the shear of the braids. Then the in-plane shear moment increased with the shear angle with a constant stiffness. There was no increase in the stiffness until the shear angle reached large values. This was due to the fact that the decrease in the width of unit cell was taken into account in the shear moment calculation. A higher temperature above melting point involved a lower shear moment (0.0175 mm N at 19.5°, 0.00498 mm N at 41.6° and 0.00192 mm N at 38.2°, respectively, for 170 °C, 180 °C, and 190 °C), with the better lubricant effect among the tows. While a greater displacement rate conducted a greater shear moment (0.00498 mm N at 41.6°, 0.00841 mm N at 36.3°, and 0.0125 mm N at 27.3°, respectively, for 25/50/100 mm/min). The greater rate drove greater viscosity, therefore, the moment was greater at the same time the greater rate accelerated the in-plane shear behavior, which means the locking angle was smaller. These experimental results show the dependences of temperature and displacement rate on the in-plane shear behavior of braided thermoplastic preform, which is important for the forming simulation.

The following parts experimentally investigated the influences of unit cell geometry parameters on the in-plane shear behavior by applying the analytical model at an elevated temperature of 180 °C with constant displacement rate of 25 mm/min; the corresponding bias-extension tests results of the set of 6 different specimens are listed in Table 3. By comparing global load, displacement, and shear angle during the extension test, shear moment versus shear angle curves can be finally obtained. The shear moment is significant since it is the value that is conjugated to the unit cell geometry.

### 4.2. Influence of Braiding Angle on Thermal In-Plane Shear Behavior

**Figure 11:**
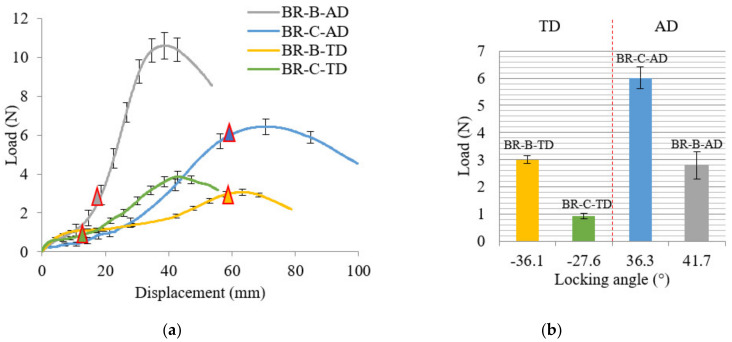
Influence of the braiding angle on thermal in-plane shear behavior: (**a**) global load versus displacement curves and (**b**) global load at locking angle.

The influence of braiding angle on thermal in-plane shear behavior is presented as global load versus displacement curves at first, shown in Figure 11a. The beginning of the curve is the rigid body motion of the in-plane shear stage, and it finishes until the triangle mark, where the locking angle occurs. The load at the locking angle is presented in Figure 11b. BR-C-AD, with a larger intertow angle in the extension direction (AD) was compared with BR-B-AD; and BR-B-TD with a larger intertow angle in the extension direction (TD) was compared with BR-C-TD; they possessed same tow waviness ratio and cover factor. The former ones with the larger intertow angle in the extension direction performed a larger displacement to obtain a locking angle during the rotation field, and finally, a greater global load was produced (the mean value of the extension loads for AD and TD directions rises respectively by 2.2 and 3.3 times with the intertow angle increasing from 70° to 110°).

**Figure 12:**
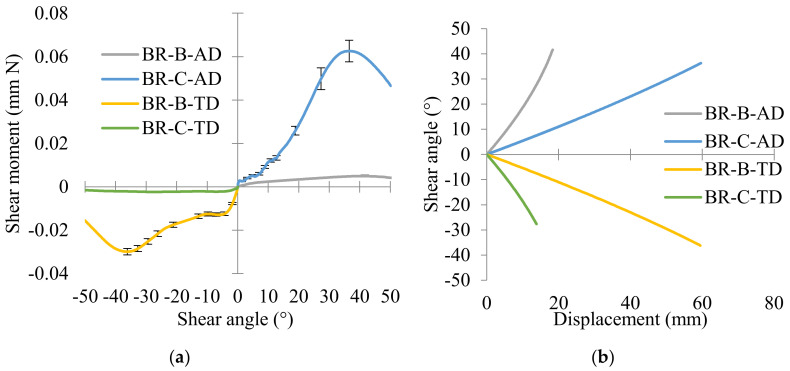
Influence of braiding angle on thermal in-plane shear behavior: (**a**) shear moment versus shear angle curves and (**b**) shear angle versus displacement curves.

The shear moment versus shear angle curves based on the unit cell with different braiding angles are presented in Figure 12a, with the shear moment in the function of the shear angle. It is the same situation as the load versus shear angle curves; for the extension tests in the same direction, the mean value of the shear moments for AD and TD directions rises, respectively, by 13.3 and 13.1 times with the intertow angle increases from 70° to 110°. What is more, for the same braids, when the braiding angle is greater than 45° (55°), it is easier to deform in the AD direction than in the TD direction. The shear moment value of BR-C-AD (reached 0.061mm N) is greater than that of BR-C-TD (−0.0023 mm N). The braids with an obtuse inter-tow angle in AD were easily conducted the shear behavior to an acute inter-tow angle, therefore performing greater shear moment. When the braiding angle is less than 45° (35°), it is easier to deform in the TD direction than in the AD direction, and the value (−0.03 mm N) of BR-B-TD is greater than that of BR-B-AD (0.0046 mm N).

The analysis of shear angle versus displacement curves shown in Figure 12b is essential to obtain better knowledge about the correlation between braiding angle, shear angle, and shear moment. From the experimental results, it could be concluded that the displacement at locking angle is only related to the intertow angle in the extension direction (larger intertow angle led to a larger displacement); but whether the intertow is an obtuse or acute angle, the locking angle is always around 30–40°. According to the textile deformability study of Lomov et al. [17], an in-plane shear stage includes the tow rotation field and transverse compression field. That means that exceeding a limit shear angle value (locking angle), the relative displacement of the in-plane shear stage is no longer a simple rotation field but added by a transverse compression field; the shear moment at the locking angle reaches the greatest value. BR-C-AD and BR-B-TD possess an obtuse intertow angle (110°) in the extension direction, the intertow angle decreased from 110° to 70–80° to reach the maximum shear moment. The displacement of this rotation field was long (around 60 mm) and then the experiment almost ended. BR-B-AD and BR-C-TD possess an acute intertow angle (70°) in the extension direction, the intertow angle decreased from 70° to 30–40° under the extension to reach the maximum shear moment, where the displacement was about 10–20 mm, and then began to bear the tension stress until the end of the experiment. The reach of the max shear moment means the end of the rotation field of the in-plane shear stage. The shear moment strongly depends on the rotation rate $\frac{\dot{u}}{\dot{\gamma }}$, which has been included in Equation (13). From Figure 12b, the $\frac{\dot{u}}{\dot{\gamma }}$ (obtuse angle) > $\frac{\dot{u}}{\dot{\gamma }}$ (acute angle), a larger angle performs a larger rotation rate and a greater deform capacity, that is why the shear moment is greater.

(13) $$F\frac{\dot{u}}{\dot{\gamma }}=M_{s}\left(\gamma \right)\frac{S_{C}}{S_{u}}+\frac{1}{2}M_{s}\left(\frac{\gamma }{2}\right)\frac{S_{B}}{S_{u}}$$

### 4.3. Asymmetry of the Thermal In-Plane Shear Behavior in AD and TD

**Figure 13:**
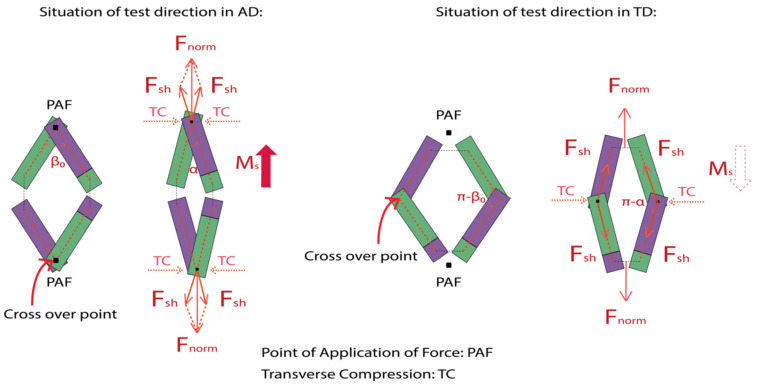
Scissor mechanisms in AD and TD for in-plane shear behavior.

For this situation, BR-B-AD is compared to BR-C-TD, and BR-C-AD is compared to BR-B-TD, with the same intertow angle in extension direction, same tow waviness, and cover factor. It can be noticed that at the locking angle, global force at the locking angle is always greater and the shear moments are almost two times greater than those in TD. For a given intertow angle, in-plane shear behavior in AD is always better than that in TD; it is necessary to analyze the deformation scissor mechanism in AD and TD for better knowledge. Reaching the locking angle is an important moment, where the in-plane shear behavior switch from simple rotation field to transverse compression added field, Figure 13 shows the scissor mechanisms in AD and TD approaching the switching time. The points of application of force applied to the cross-over points in AD but not in TD. When approaching the switching time, the AD specimen and TD specimen still possess the same intertow angle. Once upon the transverse compression is added, it causes the upper and lower scissors in AD to continue to reduce the intertow angle, thus, the shear angle continues to increase; while in TD, the transverse compression causes the left and right scissors to break at the cross over point, the intertow angle will not decrease anymore. Wrinkling or slippage will occur without changing the angle, depending on the width of specimen. That is why the locking angle in AD is always larger than TD for a given intertow angle, leading to a greater force and a greater moment.

### 4.4. Influence of Tow Waviness and Cover Factor on Thermal In-Plane Shear Behavior

**Figure 14:**
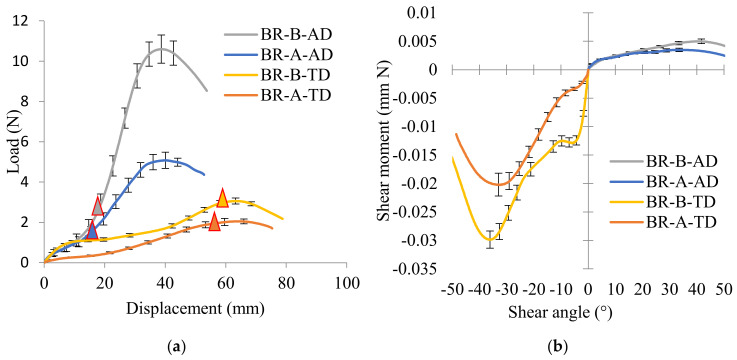
Influence of tow waviness and cover factor on thermal in-plane shear behavior: (**a**) load versus displacement curves, and (**b**) shear moments versus shear angle curves.

The influence of tow waviness and cover factor on thermal in-plane shear behavior is investigated on the BR-A and BR-B specimens with the same braiding angle. Global load versus displacement curves and shear moment versus shear angle curves are shown in Figure 14. In the same extension direction, since the intertow angle is the same, the displacement at locking angle is basically the same, where the triangles are marked in Figure 14a. The curves possess a similar profile; the BR-B specimens with larger tow waviness and cover factor performed a larger force at the locking angle (1.8 and 1.6 times greater, respectively, in the AD and TD directions). Same results for the shear moment versus shear angle curves in Figure 14b, in the same extension direction, the larger tow waviness ratio and cover factor BR-B specimens performed greater in-plane shear moment value (1.3 and 1.5 times greater respectively in AD and TD directions). Comparing the same braids (BR-A-AD and BR-A-TD; BR-B-AD and BR-B-TD), the intertow angle in TD is an obtuse angle, thus the deformation capacity in TD is greater than that in AD, the load at locking angle and max shear moment in TD are greater than those in AD.

**Figure 15:**
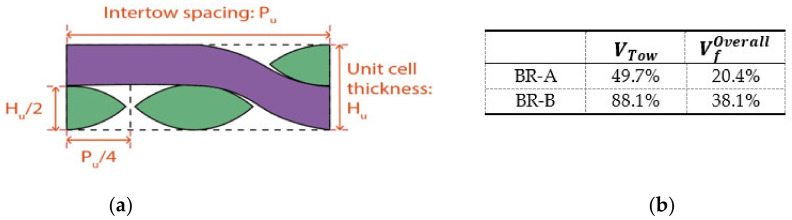
(**a**) Cross-section of the unit cell and (**b**) tow volume fraction and fiber volume fraction of the braided prepregs.

Tow waviness and cover factor as another two-unit cell parameters for braids were studied together in this part, since they are related to overall fiber volume fraction $V_{f}^{Overall}$, which is important for the deformation simulation of braided prepregs in thermal condition. It depends on the tow fiber volume fraction in the tow $V_{f}^{Tow}$ and the tow volume fraction $V_{Tow}$ (Equation (14)). $V_{Tow},$ measuring how much volume the tow occupies in the entire braided unit cell, has been described in the study of Tang et al. [47]. Hereby it is related to the cover factor (surface ratio $S_{Tow}$) and tow waviness (cross-sectional height ratio $H_{Tow}$) (Equation (15)). The illustration of unit cell cross-section is presented in Figure 15a, from which $H_{Tow}$ is obtained. With the measurement of unit cell geometry parameters and flax/PP hybrid yarn volume fraction (41/59), the $V_{Tow}$ and $V_{f}^{Overall}$ calculated by Equations (17) and (14) are listed in Figure 15b for the BR-A and BR-B braids.

(14) $$V_{f}^{Overall}=V_{f}^{Tow}\times V_{Tow}$$

(15) $$V_{Tow}=S_{Tow}\times H_{Tow}$$

(16) $$H_{Tow}=\frac{H_{u}}{2}/\frac{P_{u}}{4}$$

(17) $$V_{Tow}=CF\times 2WR$$

According to the tow waviness and cover factor, the unit cell 2D biaxial braided prepregs in thermal condition can be simulated by calculating the thickness and surface distributions of the unit cell, meaning the thermal impregnation distribution. Taking BR-A as an example, the numeric matrix is given as below; the finite element analysis needs to be done to obtain an accurate simulation.

(18) $$X=\left(\begin{array}{ccccccc} 0 & \frac{W_{t}}{2\cos\frac{\beta }{2}} & \frac{W_{u}}{4} & \frac{W_{u}}{2}-\frac{W_{t}}{2\cos\frac{\beta }{2}} & \frac{W_{u}}{2} & \cdots & W_{u} \end{array}\right)$$

(19) $$Y=\left(\begin{array}{ccccccc} 0 & \frac{W_{t}}{2\sin\frac{\beta }{2}} & \frac{L_{u}}{4} & \frac{L_{u}}{2}-\frac{W_{t}}{2\sin\frac{\beta }{2}} & \frac{L_{u}}{2} & \cdots & L_{u} \end{array}\right)$$

(20) $$Z=\left[\begin{array}{ccccccc} H_{u} & 0 & 0 & 0 & H_{u} & \ldots & H_{u}\\ 0 & \frac{H_{u}}{2} & 0 & \frac{H_{u}}{2} & 0 & \ldots & 0\\ 0 & 0 & H_{u} & 0 & 0 & \ldots & 0\\ 0 & \frac{H_{u}}{2} & 0 & \frac{H_{u}}{2} & 0 & \ldots & 0\\ H_{u} & 0 & 0 & 0 & H_{u} & \ldots & H_{u}\\ \vdots & \vdots & \vdots & \vdots & \vdots & \ddots & \vdots \\ H_{u} & 0 & 0 & 0 & H_{u} & \ldots & H_{u} \end{array}\right]$$

**Figure 16:**
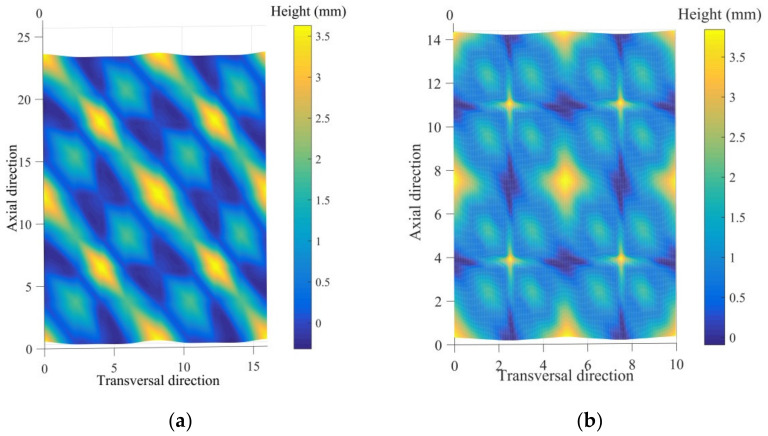
Thermal impregnation distribution of 2D biaxial braided prepregs unit cell: (**a**) BR-A and (**b**) BR-B.

The final simulation of the unit cell of BR-A and BR-B braided prepregs in the thermal condition is shown in Figure 16. It can be clearly observed that the BR-B with a larger fiber volume fraction $V_{f}^{Overall}$ (38.1%), performed better thermal impregnation with less void volume. In one unit cell, BR-B specimens always performed greater in-plane shear stiffness, presented greater maximum shear moment in both AD and TD than BR-A specimens. The larger in-plane shear stiffness led to a larger locking angle, whether in AD or TD. A small fiber volume fraction $V_{f}^{Overall}$ indicates that the void volume is large when a certain shear angle was reached, the structure dispersed and failed to continue the in-plane shear behavior, so the displacement and shear angle were both small. This means that it is very important to control the tow waviness and cover factor during the braiding process to obtain compact reinforcements with a larger overall fiber volume fraction.

## 5. Conclusions

The thermomechanical non-symmetric in-plane shear behavior of 2D biaxial braided preform is deeply investigated in this present study by applying the analytical model of extension based on unit cell geometry in an elevated temperature chamber. The thermo-condition dependent experiments were preliminarily carried out and showed that higher temperature and lower displacement rate led to a lower in-plane shear moment. Secondly, the influence of unit cell geometry parameters on thermal in-plane shear behavior was studied. It showed that the braided preform with a larger intertow angle with a great rotation rate was easily deformed with a greater in-plane shear moment in the bias-extension direction. The lower tow waviness and cover factor with lower fiber volume fraction braided preform showed weak shear behavior by presenting thermal impregnation distribution simulation. What is more, the in-plane shear behaviors were completed with the experiments both in an axial direction and transversal direction due to the special asymmetric braiding structure. Scissor mechanisms were analyzed at switching time, showing that the locking angle in AD is always larger than TD for a given intertow angle, leading to a greater force and a greater moment. Finally, in Appendix A, the theoretical and experimental comparison showed a good agreement before reaching the locking angle, meaning that the analytical model can be applied at thermal condition.

As the most important deformation mode of textile reinforcements, it is very important for the thermoforming simulation to study the thermal in-plane shear behavior of 2D biaxial braided preform. Further study about shear stress and specimen dimension will be investigated. The experimental results provided good notice for textile reinforcement design and manufacturing condition decisions to better understand the deformation mode and avoid the defects formation.

## Figures and Tables

**Table 1:** The main properties of one-layer braided preform specimens.

	**BR-A-AD**	**BR-A-TD**	**BR-B-AD**	**BR-B-TD**	**BR-C-AD**	**BR-C-TD**
Braiding angle (°)	35	35	35	35	55	55
Intertow angle in the test direction (°)	70	110	70	110	110	70
Unit cell width (mm)	16.4	23.4	11.5	16.4	16.4	11.5
Tow waviness ratio	0.31	0.31	0.46	0.46	0.46	0.46
Cover factor (%)	80.1	80.1	95.7	95.7	95.7	95.7
Thickness (mm)	3.62 ± 0.03	3.62 ± 0.03	3.82 ± 0.07	3.82 ± 0.07	3.81 ± 0.09	3.81 ± 0.09
Areal density (g/m^2^)	928 ± 30	928 ± 30	1078 ± 10	1078 ± 10	1070 ± 18	1070 ± 18
Air permeability (l/m^2^/s)	859 ± 46	859 ± 46	460 ± 26	460 ± 26	471 ± 26	471 ± 26

**Table 2:** Thermo-condition variants for the extension test.

	T (°C)	V (mm/min)
Variant of T with fixed V	170	25
	180	
	190	
Variant of V with fixed T	180	25
		50
		100

**Table 3:** Extension test results of specimens at thermo-condition: 180 °C and 25 mm/min.

	Max Extension Load (N)	Displacement at Max Load (mm)	Locking Angle (°)	Displacement at Locking Angle (mm)	Load at Locking Angle (N)	Max Shear Moment (mm N)
BR-A-AD	5.08 ± 0.4	40.06 ± 2.54	33	15.77	1.56	0.0035
BR-A-TD	2.02 ± 0.18	59.66 ± 3.12	−32.5	54.33	1.91	−0.02
BR-B-AD	10.6 ± 0.7	38.7 ± 2.09	41.7	18.37	2.79	0.0046
BR-B-TD	3.06 ± 0.15	63.28 ± 4.17	−36.1	59.45	3.00	-0.03
BR-C-AD	6.43 ± 0.4	70.62 ± 4.58	36.3	59.62	6.01	0.061
BR-C-TD	3.86 ± 0.3	42.98 ± 2.51	−27.6	13.81	0.92	−0.0023

**Figure A1:**
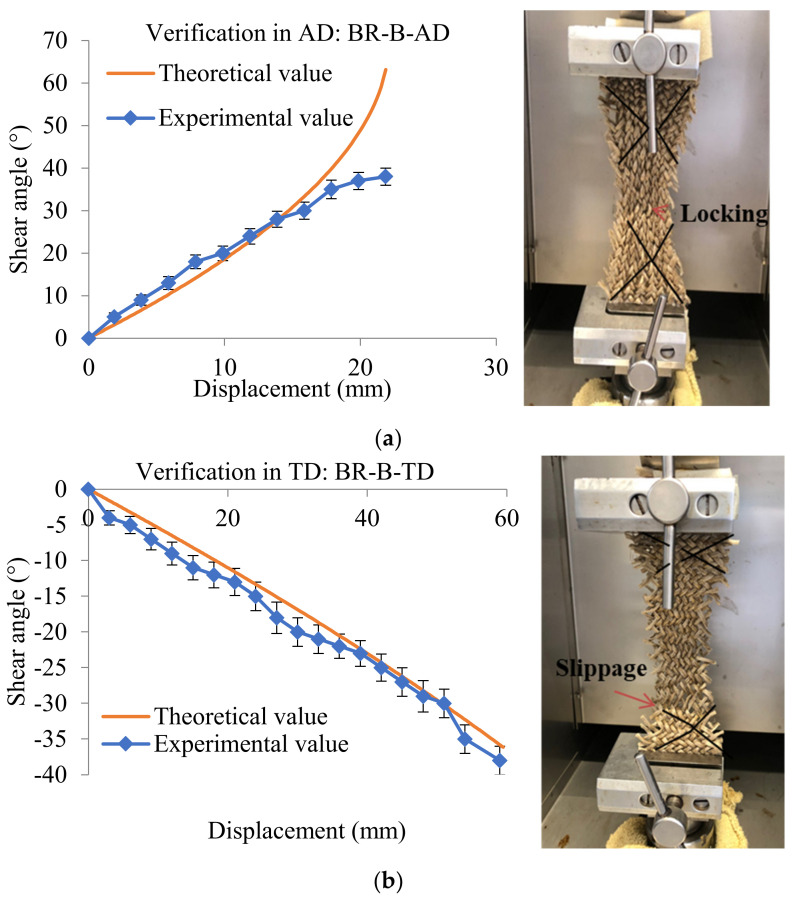
Thermo-conditional verification of analytical model: (**a**) BR-B-AD and (**b**) BR-B-TD.

## Data Availability

Data are contained within the article.

## Appendix A. Thermo-Conditional Verification of Analytical Model

The experimental in-plane shear angles in zone C were obtained by optical measurements during the bias-extension test on specimens BR-B-AD and BR-B-TD, as representative braided specimens, and compared with the theoretical values to verify the analytical model. Thermo-condition was determined at a temperature of 180 °C with a constant displacement rate of 25 mm/min. The verification of the theoretical model presented in Equation (7) needed to be conducted in both AD and TD directions. The comparison of the theoretical and experimental shear angle values in zone C for BR-B-AD and BR-B-TD (braiding angle 35°) is shown in Figure A1. It can be clearly observed that the theoretical curve is consistent with the experimental data for BR-B-AD until separation appears approximately at 40° shear angle. This is correct, and it is further confirmed that before the separation, braided tows sustained rigid body motion (rotation + translation of the center) [17] of the in-plane shear stage until the locking angle about 40° reached (the value of locking angle depends on the tow width), then the tows started to bear the tension stress until test finished. For the verification of BR-B-TD, shear angles are negative values, the theoretical curve is consistent with the experimental data until the maximum shear angle. It was also confirmed that the slippage phenomena appeared after the maximum shear angle and the test had finished. The images on the right side of Figure 11 show the specimen after reaching the locking angle. The three different zones of the theoretical kinematics of the test (presented in Figure 3) are clearly observed in the deformed configuration. With the load application, the locking angle was reached, which made the tows embossment, and wrinkling was observed for BR-B-AD; while the tow slippage was observed for BR-B-TD. The comparison shows good agreement in both AD and TD direction, which is enough to verify the accuracy of theoretical derivation and it can be confirmed that the analytical model can be applied in the thermo-condition. The matrix is weak in thermo-state above the melt temperature and does not modify the kinematics of the test, which only modifies the in-plane shear stiffness.
